# Monitoring of breast cancer progression via aptamer-based detection of circulating tumor cells in clinical blood samples

**DOI:** 10.3389/fmolb.2023.1184285

**Published:** 2023-06-08

**Authors:** Olga S. Kolovskaya, Alena V. Zyuzyukina, Justin P. Dassie, Galina S. Zamay, Tatiana N. Zamay, Nina V. Boyakova, Vladimir A. Khorzhevskii, Daria A. Kirichenko, Ivan N. Lapin, Irina A. Shchugoreva, Polina V. Artyushenko, Felix N. Tomilin, Dmitry V. Veprintsev, Yury E. Glazyrin, Zoran Minic, Vladimir K. Bozhenko, Elena A. Kudinova, Yana Y. Kiseleva, Alexey V. Krat, Eugene V. Slepov, Anton S. Bukatin, Ruslan A. Zukov, Pavel A. Shesternya, Maxim V. Berezovski, Paloma H. Giangrande, Anna S. Kichkailo

**Affiliations:** ^1^ Laboratory for Biomolecular and Medical Technologies, Prof. V.F. Voino-Yasenetsky Krasnoyarsk State Medical University, Krasnoyarsk, Russia; ^2^ Laboratory for Digital Controlled Drugs and Theranostics, Federal Research Center “Krasnoyarsk Science Center of the Siberian Branch of the Russian Academy of Science”, Krasnoyarsk, Russia; ^3^ Department of Oncology and Radiation Therapy, Faculty of Medicine, Prof. V.F. Voino-Yasenetsky Krasnoyarsk State Medical University, Krasnoyarsk, Russia; ^4^ Krasnoyarsk Regional Clinical Cancer Center Named After A.I. Kryzhanovsky, Krasnoyarsk, Russia; ^5^ Department of Internal Medicine, University of Iowa, Iowa, IA, United States; ^6^ Department of General Surgery, Named After Prof. M.I. Gulman, Faculty of Medicine, Prof. V.F. Voino-Yasenetsky Krasnoyarsk State Medical University, Krasnoyarsk, Russia; ^7^ Department of Pathological Anatomy, Faculty of Medicine, Prof. V.F. Voino-Yasenetsky Krasnoyarsk State Medical University, Krasnoyarsk, Russia; ^8^ Krasnoyarsk Regional Pathology-Anatomic Bureau, Krasnoyarsk, Russia; ^9^ Laboratory of Advanced Materials and Technology, Siberian Physical Technical Institute, Tomsk State University, Tomsk, Russia; ^10^ School of Non-Ferrous Metals and Materials Science, Siberian Federal University, Krasnoyarsk, Russia; ^11^ Laboratory of Physics of Magnetic Phenomena, Kirensky Institute of Physics, Krasnoyarsk, Russia; ^12^ Department of Chemistry and Biomolecular Sciences, University of Ottawa, Ottawa, ON, Canada; ^13^ Russian Scientific Center of Roentgenoradiology, Moscow, Russia; ^14^ Alferov Federal State Budgetary Institution of Higher Education and Science, Saint Petersburg National Research Academic University of the Russian Academy of Sciences, Saint Petersburg, Russia; ^15^ Institute for Analytical Instrumentation of the Russian Academy of Sciences, Saint Petersburg, Russia; ^16^ Platform Discovery Sciences, Biology, Wave Life Sciences, Cambridge, MA, United States

**Keywords:** RNA-aptamer, MDA-MB-231, breast cancer, molecular subtypes, circulating tumor cells, mammaglobin

## Abstract

**Introduction:** Breast cancer (BC) diagnostics lack noninvasive methods and procedures for screening and monitoring disease dynamics. Admitted CellSearch^®^ is used for fluid biopsy and capture of circulating tumor cells of only epithelial origin. Here we describe an RNA aptamer (MDA231) for detecting BC cells in clinical samples, including blood. The MDA231 aptamer was originally selected against triple-negative breast cancer cell line MDA-MB-231 using cell-SELEX.

**Methods:** The aptamer structure in solution was predicted using mFold program and molecular dynamic simulations. The affinity and specificity of the evolved aptamers were evaluated by flow cytometry and laser scanning microscopy on clinical tissues from breast cancer patients. CTCs were isolated form the patients’ blood using the developed method of aptamer-based magnetic separation. Breast cancer origin of CTCs was confirmed by cytological, RT-qPCR and Immunocytochemical analyses.

**Results:** MDA231 can specifically recognize breast cancer cells in surgically resected tissues from patients with different molecular subtypes: triple-negative, Luminal A, and Luminal B, but not in benign tumors, lung cancer, glial tumor and healthy epithelial from lungs and breast. This RNA aptamer can identify cancer cells in complex cellular environments, including tumor biopsies (e.g., tumor tissues vs. margins) and clinical blood samples (e.g., circulating tumor cells). Breast cancer origin of the aptamer-based magnetically separated CTCs has been proved by immunocytochemistry and mammaglobin mRNA expression.

**Discussion:** We suggest a simple, minimally-invasive breast cancer diagnostic method based on non-epithelial MDA231 aptamer-specific magnetic isolation of circulating tumor cells. Isolated cells are intact and can be utilized for molecular diagnostics purposes.

## Graphical Abstract

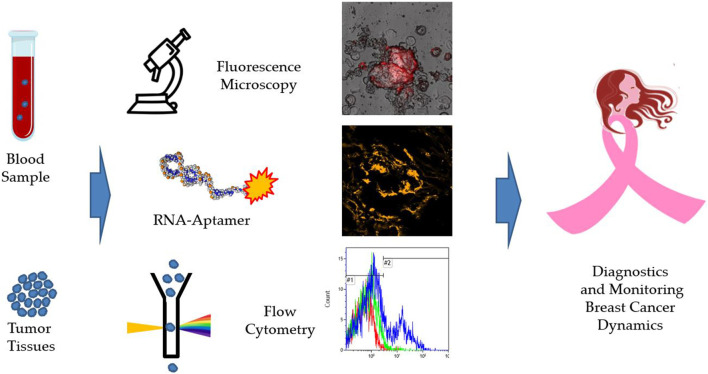

## 1 Introduction

Breast cancer (BC) occupies a leading position in the structure of the incidence of malignant neoplasms. Despite treatment advances, the mortality rate remains high and approximates 40% (Ferlay et al., 2013). It is associated with metastasis that occurs in the later stages. Standard BC diagnostics still use tissue biopsy, an invasive procedure; however, screening the population requires simple and minimally invasive diagnostic methods. Biomarkers detection in blood plasma, also called liquid biopsy, is one of the preferred approaches for BC diagnosis. Currently, the CellSearch^®^ is the only Food and Drug Administration-approved method that can be used to monitor breast, prostate, and colorectal cancer patients. Its isolation is based on anti-epithelial cell adhesion molecule (EpCAM) antibodies for the positive capture of tumor cells of epithelial origin. CTCs can be stained by cytokeratin, CD45 (typical lymphocyte antigen), and DAPI for semi-automated morphologic analysis at a single-cell level. However, epithelial cell marker-dependent criteria are not ideal because it does not allow the detection of cell mesenchymal phenotype (Lozar et al., 2020).

Today, the recommended biomarker for detecting BC in the blood is cancer antigen 15-3 (CA 15-3). An increased preoperative level of CA 15-3 was found to be a predictor of the rapid development of BC (Mudduwa et al., 2018). Elevated preoperative levels of CA 15-3 and the cancerous-embryonic antigen (CEA) are interrelated but have independent prognostic significance since, in some cases, the results obtained based on data on the levels of these markers are inconsistent (Shao et al., 2015). However, another evidence suggests that CA 15-3 is not a strictly specific biomarker for BC, as it may appear in ovarian cancer (Dolscheid-Pommerich et al., 2017) and endometrium (Tas and Yavuz, 2017).

DNA or RNA aptamers selected for whole cells or tissues without prior knowledge of the target can be used for new cancer biomarker discovery (Zamay et al., 2015). Aptamers to several protein biomarkers of BC have been described in the literature: epidermal growth factor 2 (HER2) (Liu et al., 2012), CA 15-3 (Agnihotri et al., 2014).

Here we present an RNA aptamer (MDA231) selected against the MDA-MB-231 cell line originating from malignant triple-negative breast cancer. This aptamer demonstrates the sensitivity to oncogenic proteins on tumor cells in cancer tissues, margins, distant tissues, lymph nodes, and circulating tumor cells (CTCs) in real clinical samples of BC patients with different molecular subtypes. Tissue samples from 27 patients were used to prove the binding of this aptamer to different molecular subtypes. CTCs were isolated from blood samples of 22 patients with BC taken before the surgery and for 7 patients 32 months after the surgery.

## 2 Materials and methods

The study was approved by the Local Ethics Committee KGBUZ “Krasnoyarsk regional clinical oncological dispensary named after A.I. Kryzhanovsky” protocols # 8/2016 dated March 16, 2016, and #55 dated December 28, 2022.

### 2.1 Molecular modeling

The secondary structure of the aptamer was predicted using the mFold program (Zuker, 2003) in the presence of ions Na^+^ 146 mM and Mg^2+^ 0.5 mM at 4°C. The corresponding spatial structure was modeled with SimRNA (Boniecki et al., 2016) and VMD programs (Humphrey et al., 1996; Jeddi and Saiz, 2017). Molecular dynamics (MD) simulations of 200 ns were carried out using GROMACS 2019.6 package (Abraham et al., 2015) with the Amber14sb force field (Maier et al., 2015). The TIP3P model (Jorgensen et al., 1983) for water was used. The negative charge of the aptamer was neutralized with Na^+^ ions. Then, Na^+^ and Cl^−^ ions at the physiological concentration (0.15 M) were added to the systems. The velocity rescaling thermostat (Bussi et al., 2007) was used to keep the system at a temperature of 310 K in combination with the Parrinello-Rahman barostat (Parrinello and Rahman, 1981) to keep the pressure at 1 bar. The most representative structure of the aptamer was obtained by the clustering analysis of MD trajectories with the quality threshold algorithm (Heyer et al., 1999) implemented in VMD. The RMSD of the phosphorus atoms was used as a metric function; the cutoff was 0.5 nm.

### 2.2 Flow cytometry of clinical tissue samples

The affinity and specificity of the evolved aptamers were evaluated by flow cytometry on an FC-500 Flow Cytometer (Beckman Coulter Inc., United States). Breast cancer tissues, margin and distant tissues, and lymph nodes were washed with DPBS. Next, tissues were minced by a blade and pipetted with DPBS to obtain a homogeneous solution. The cell suspension was filtered through 70 μm filters; obtained cells were centrifuged at 3,000 g for 5 min and washed three times with DPBS. Next, cells were pre-incubated with yeast RNA (1 ng μL^−1^) for 30 min and then with 70 nM of Cy3-labeled aptamer MDA231 for 30 min at 25°C with shaking. Each sample contained 3 × 10^5^ cells. BC cells pre-incubated with 1 ng μL^−1^ yeast RNA and 70 nM Cy3-labeled (AG) 40-oligonucleotide were used as a control. To determine the dissociation constant of the aptamer, a series of measurements of the fraction of bound cells depending on the aptamer concentration was performed. The hyperbolic regression model corresponding to the Michaelis-Menten equation was fitted to the set of obtained data, from which Kd was determined.

### 2.3 Isolation of CTCs from human blood

Isolation of CTCs was performed from 3.5 ml of patients’ blood 1–1.5 h after collecting into BD Vacutainer Heparin Tubes. The blood was centrifuged (1.500 g for 10 min) to remove the plasma. The cell pellet was transferred into a 15 ml centrifuge tube using a BSA-treated tip. Red blood cells (RBCs) were lysed with hypotonic NH_4_Cl solution in a Vacutainer tube. Cells were rinsed with 2 ml of 0.42% NH_4_Cl with heparin, pipetted up and down 5 times, and poured into the 15 ml tube with 8 ml of 0.42% NH_4_Cl with heparin, incubated for 10 min on a shaker and centrifuged at 3.500 g for 5 min. The remaining cell pellet was resuspended in 100 μl DPBS and incubated with masking yeast RNA (0.1 ng μL^−1^) for 30 min at room temperature to reduce nonspecific binding. The sample was centrifuged at 3.500 g for 5 min. The supernatant was removed and incubated for 30 min with 100 µg of streptavidin-coated paramagnetic beads (Promega Corporation, United States) functionalized with 100 nM biotinylated aptamer MDA231. Cells bound with magnetic beads via the aptamers were pulled on a tube wall using a magnetic stand, resuspended in 100 μl of DPBS buffer, and concentrated by a magnet. The pellet containing CTCs was preincubated with masking RNA 0.1 mg/ml in cold DPBS with 0.1% BSA for 30 min to exclude non-specific binding. Afterward, the mixture was stained with a Cy3-labeled aptamer at a final concentration of 70 nM and FITC-labeled anti-cytokeratin antibodies (final concentration 0.001 mg/ml) for 30 min. After staining, the cell pellet was spread evenly on a glass slide to prepare the smears and then fixed in methanol for 5 min, followed by staining with Romanovsky-Giemsa dye. Immunocytochemistry of the isolated CTCs was performed using a standard procedure. CTCs were placed on poly-lysine coated glass slides, and fixed in 100% methanol, permeabilized with Triton X-100; non-specific binding was blocked with 10% goat serum. For the staining, a recombinant anti-mammaglobin (Abcam) and recombinant anti-GCDFP15 antibodies (Abcam), and secondary Goat anti-Humanan IgA:HRP (Bio-Rad) were used with a dilution of 1:100. Counting of circulating tumor cells were done on a fluorescent microscope Axiostar plus (Carl Zeiss Group, Germany).

### 2.4 RT-qPCR assay

Mammaglobin mRNA was detected using real-time qPCR, as described earlier (Bojenko et al., 2013) on a DT-96 amplifier (DNA-technology, Russia). For one PCR reaction, 10 µl was used. 2.5X reaction mixture; 1 μl. MgCl_2_ (25 mM) (Syntol, Russia), 7.5 µl of deionized water, 1 μl. Forward primer (10 pmol), 1 μl. Reverse primer (10 pmol) (Syntol, Russia), 0.5 µl primer “probe” containing the Tag man fluorophore, 5 μl. cDNA sample. The following primers were used: Forward 5′- GAA​CAC​CGA​CAG​CAG​CA-3′, Reversed 5′-TCC​AAT​AAG​GGG​CAG​CC-3′, Sample 5′-FAMTGGTCCTCATGCTGGCGGCC-3′. RNA aptamer concentration after internalization into the cells was quantified by RT-qPCR using SYBR green PCR mastermix as outlined previously (Thiel et al., 2015). Briefly, aptamer MDA231 in serum-free medium and tRNA to 100 μg/ml were incubated with the cells at 37°C for 30 min, and unbound RNA was washed out with the washing buffer. RNA was extracted using TRIzol. Control Sel1 RNA was added to lysed cells containing 5 × 10^−3^ pmol/ml. The recovered RNA was quantified by RT-qPCR using SYBR green PCR mastermix as appropriate during the PCR. The Sel1 PCR is used as a normalization control for sample processing.

### 2.5 Histological and tissue staining

Breast cancer tissue pieces were frozen in liquid nitrogen, sliced into 5 µm sections by Microm HM525 Cryostat, and placed on poly-lysine coated glass slides. Tissue sections were incubated with yeast RNA (1 ng μL^−1^) (Sigma Aldrich, United States) for 1 h before the aptamer’s non-specific binding and then incubated with 50 nM of Cy3-labeled aptamer MDA231 for 1 h in a humidified atmosphere and washed with DPBS. Bio Mount mounting medium (Bio-Optica, Italy) was used to fix the sections.

### 2.6 Aptamer targets identification

Protein targets of MDA231 were identified using a modified AptaBID procedure (Berezovski et al., 2008) as previously described (Zamay et al., 2014; Zamay et al., 2017). Briefly, manually shredded tissues were lysed in 0.1% Na deoxycholate solution. Potential DNA binding sites were masked by yeast RNA and non-specific DNA with poly-AG sequence, then incubated with biotinylated MDA231. Streptavidin-coated magnetic beads (Promega Corporation, United States) were used to extract the aptamer-protein complex from the solution. Then the bound proteins were detached from the complex by 8 M urea. The derived proteins were reduced by dithiothreitol, alkylated by iodoacetamide, digested by trypsin (Sigma Aldrich, United States), and then desalted by Pierce C18 pipette tips (Thermo Scientific, United States). Mass spectrometry analyses were done by UltiMate 3000 nano-UHPLC system coupled to Orbitrap Fusion mass spectrometer (Thermo Scientific, United States).

Tissues of four patients with verified breast cancer diagnoses were taken in total, the samples of each patient were prepared in four replicates, and each replicate was analyzed by mass spectrometry in 2–3 repetitions. The complete set of 44 files was processed by Proteome Discoverer 1.4 using the SEQUEST HT search engine. Membrane and extracellular proteins found highly confident in most repeated samples were taken as target candidates.

## 3 Results

### 3.1 Aptamer modeling and analyses

In the current study, we used the MDA231 RNA aptamer to recognize breast cancer cells from clinical samples specifically. The sequence of this aptamer was previously identified and published by Dassie (Dassie, 2012) with SELEX against MDA-231/LUC and MDA-MB-231 cell lines.

**FIGURE 1:**
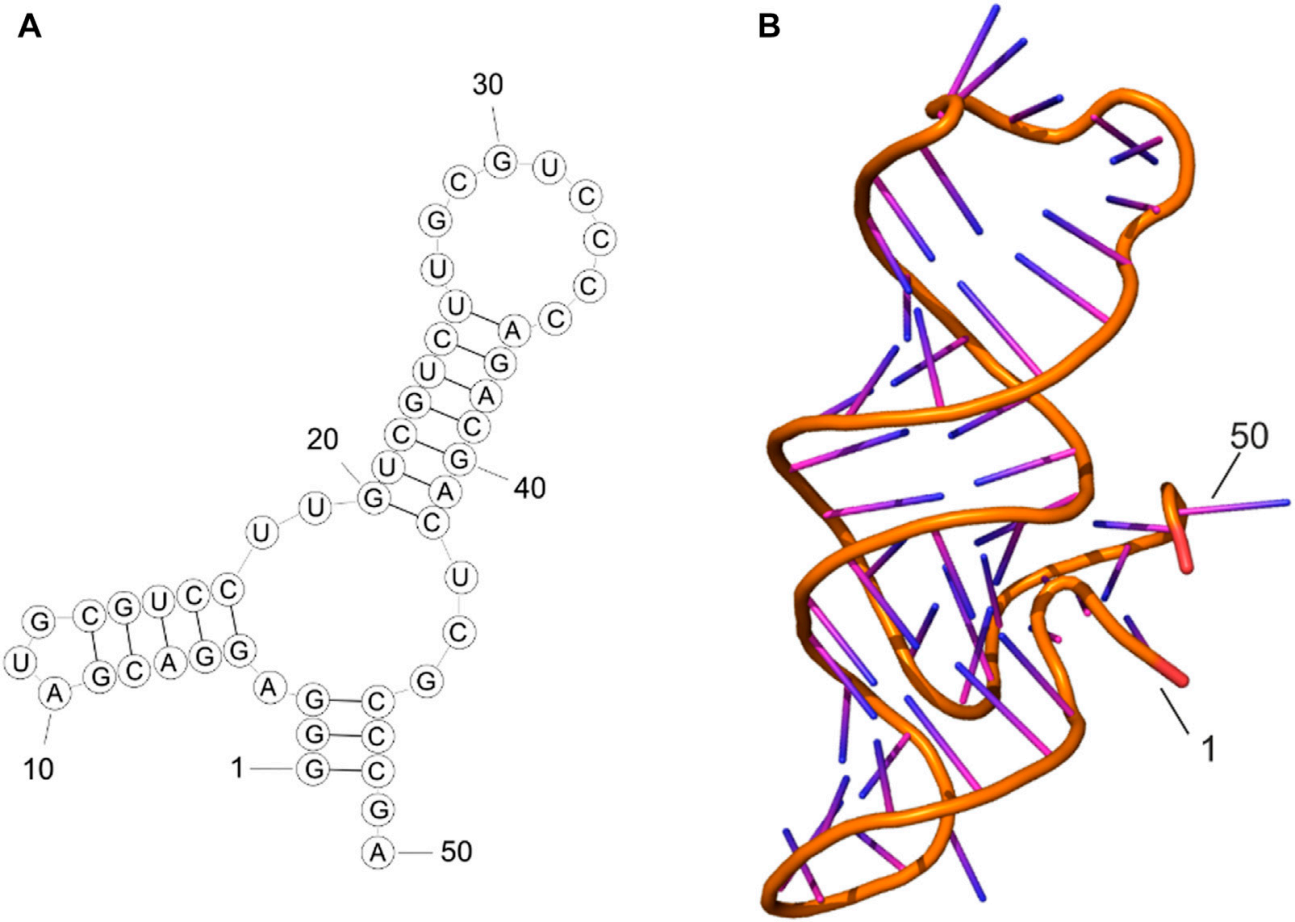
Secondary structure **(A)** and tertiary structure **(B)** of MDA231 aptamer after MD simulations.

MFold web server predicted one possible secondary structure for the aptamer MDA231 according to the sequence 5′-GGG​AGG​ACG​AUG​CGU​CCU​UGU​CGU​CUU​GCG​UCC​CCA​GA C​GAC​UCG​CC C ​GA-3′. The corresponding predicted tertiary structure was modeled using SimRNA and VMD programs. Molecular dynamic (MD) simulations followed by the clustering analysis of MD trajectories allowed us to obtain the most representative structure of the aptamer in the solution. As seen in Figure 1, aptamer forms two stem loops, the largest of which is rich in cytosines. Both stem loops remained stable throughout the 200 ns MD simulation performed under physiological conditions.

### 3.2 Aptamer specificity assessment

Aptamer MDA231’s specificity to clinical breast cancer samples was assessed by flow cytometry, laser scanning, and fluorescence microscopy. MDA231 binds in a dose-dependent manner with the cells isolated from tumor margin, distant tissue, and lymph node metastasis taken after the surgeries. The dissociation constant of 39 nM for MDA231 aptamer was the same for the patients with triple-negative and luminal A breast cancers. MDA231 at a concentration of 70 nM was used for further analyses. MDA231 did not bind with the healthy epithelial cell line MCF10A, primary cell cultures derived from healthy lung epithelial, glial brain tumor, lung cancer, and bind with breast cancer cell lines MCF7, MDA-MB-231, MDA453 (Supplementary Figure S1).

Sometimes aptamers selected against cultured cells are not very effective in clinics. Thus, we estimated the potency of this aptamer to distinguish BC cells in BC tissue clinical samples taken after the surgery.

**FIGURE 2:**
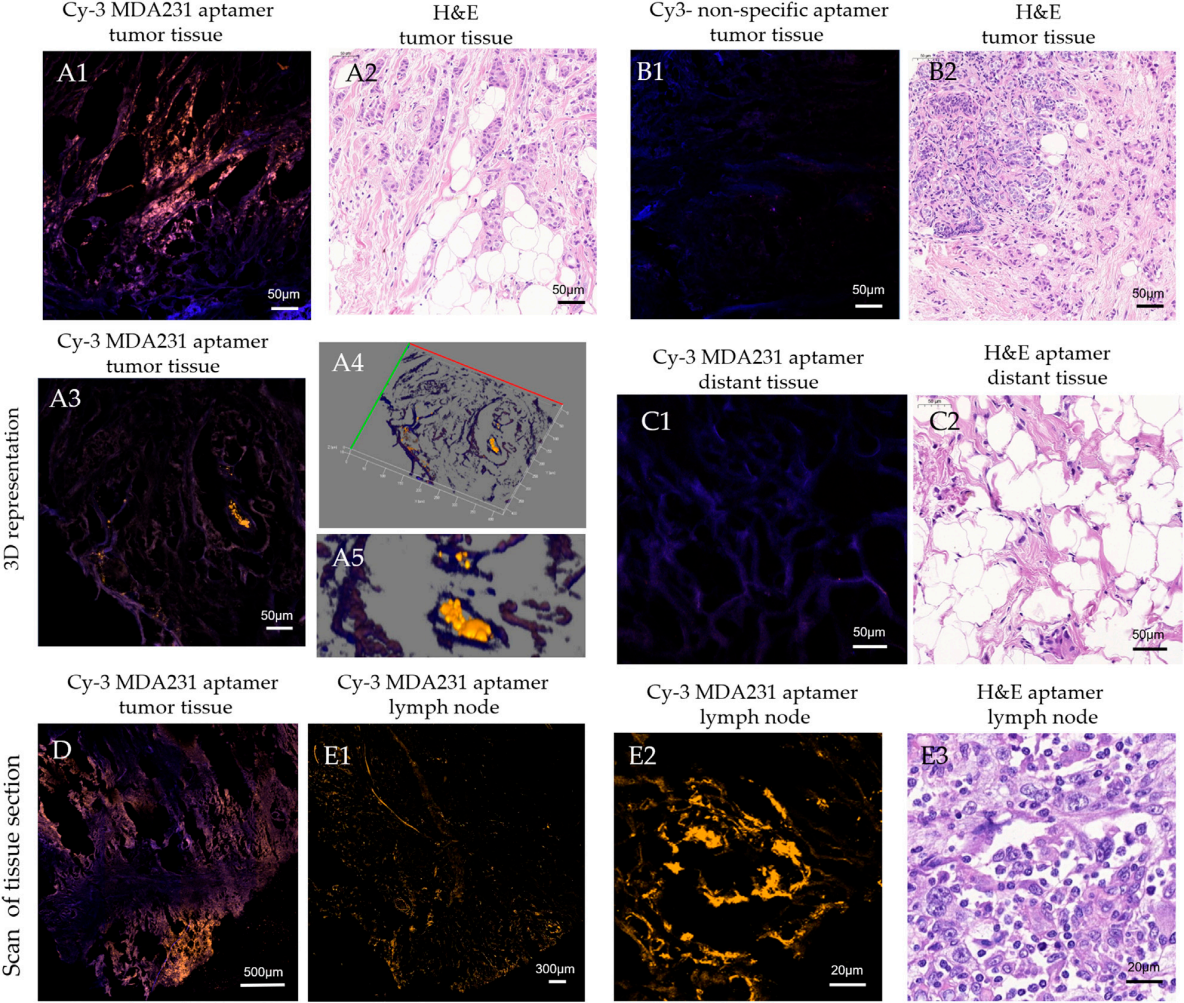
Laser scanning microscopy of histological tissue sections of breast tumor **(A, B, D)**, distant **(C)**, and metastatic lymph node **(E)** tissues stained with Cy3-labeled MDA231 aptamer [A1, A3, A4, C1, D, E1, E2], and non-specific oligonucleotide (B1). Adjacent sections were stained with hematoxylin & eosin (A2, B2, C2, E3).

MDA231 can label tumor cells in patients’ tissue samples. We evaluated the binding of the aptamer to breast cancer cells in histological tissue sections using laser scanning microscopy (Figure 2). The microscopy showed that the cells bound with MDA231 were in small clusters of 3–7, medium clusters of 8–20, and large 20–50 cells in BC tissue (Figures 2a1–a5), which matched the adjacent H&E sections (Figure 2a2). A more significant number of cancer cells were found in metastatic lymph nodes (Figures 2e1–e3). In some areas of the scanned section, MDA231 is bound with the extracellular matrix but to a lesser extent (Figure 2d). Non-specific oligonucleotide did not demonstrate any binding to cancerous cells, only slightly stained stroma (Figures 2b1, b2). No cancer cells, or very few of them stained by the aptamer, were found in the different sections of distant tissues, which was proved by H&E staining (Figures 2c1, c2). Comparing tissue sections stained with the fluorescently labeled aptamer MDA231 with hematoxylin and eosin (H&E) stained.

**FIGURE 3:**
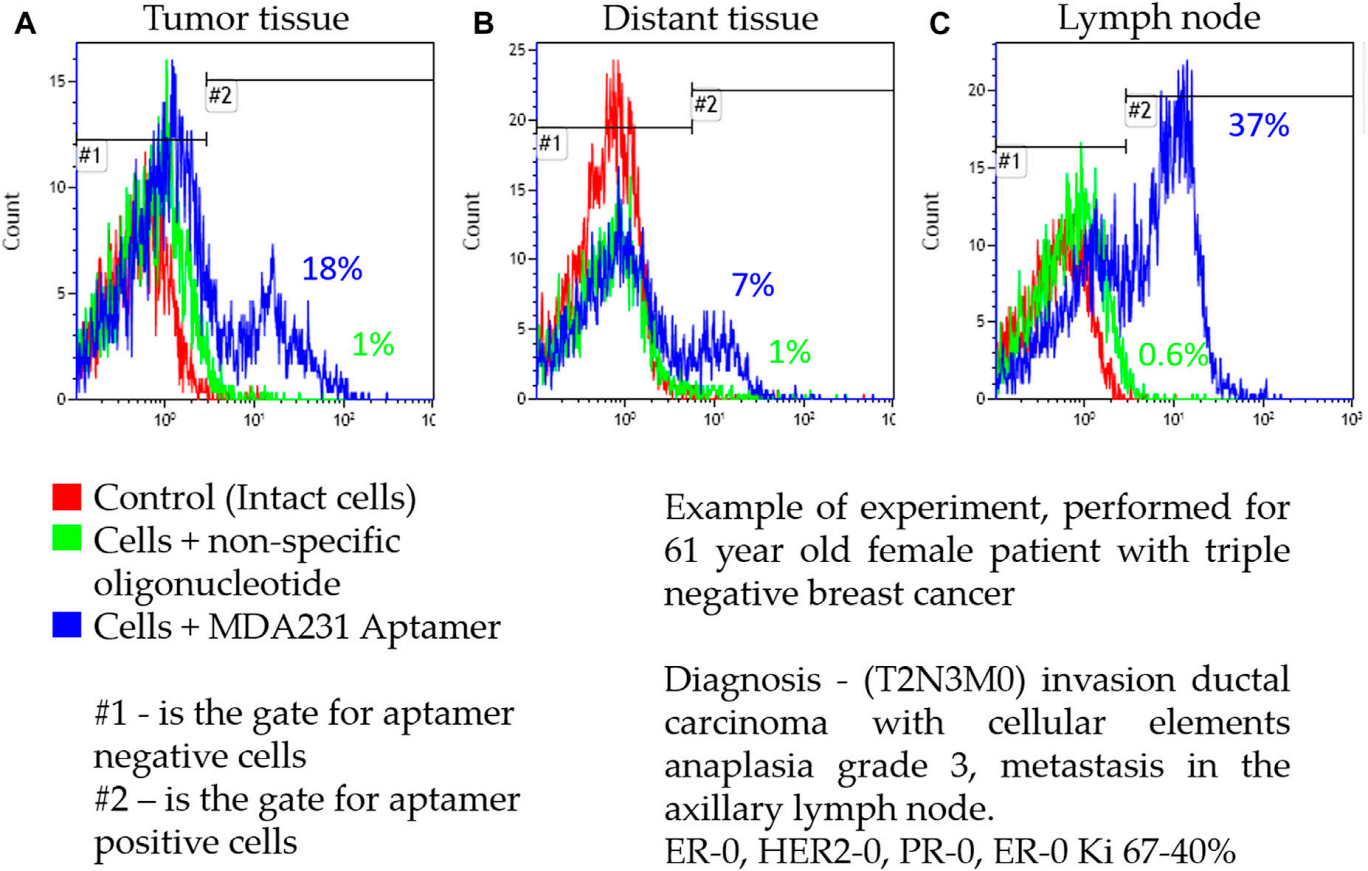
Representative flow cytometry analysis of cells isolated from tumor zone **(A)**, distant tissue **(B)**, and lymph node metastasis **(C)** from a patient with triple-negative breast cancer.

Cells isolated from the tumor zone, distant tissue, and lymph node metastasis were stained with the Cy3-labeled MDA231 aptamer. Figure 3 represents the example of flow cytometry analyses of the MDA231 aptamer binding to all cells (including malignant cells, fibroblasts, tumor stroma, etc.) derived from tumor zones, distant tissues, and lymph node metastasis from a 61-year-old female patient with triple-negative breast cancer diagnosed with invasive ductal carcinoma with cellular elements anaplasia grade 3 (T2N3M0) and metastasis in the axillary lymph node. ER-0, HER2-0, PR-0, ER-0 Ki 67%–40%. MDA 231 bound 18% of cells from tumors, 7% from distant tissues, and 37% from lymph nodes. Supplementary Table S1 presents a list of breast cancer patients included in the analysis, their age, the type and stage of the disease, and levels of molecular markers, such as HER2, PR, ER, Ki67, and disease dynamics matched with the percentage of aptamer binding.

The aptamer demonstrated different binding to all types of patient tissue probably means that the target of the aptamer is an antigen whose expression level depends on the type of tissue and the patient’s characteristics. We did not find correlations between age, grade, hormone receptor status, degree of histological differentiation, the presence of metastasis, and MDA213 binding to the cells derived from the primary tumor, margins, distant tissues, or metastasis in the lymph node. More comprehensive analyses have to be performed to find any patterns. Nevertheless, the obtained result shows that the aptamer MDA231 selected for the cell culture may be used for biomarker detection in real clinical samples. Despite that, no reliable patterns were found between aptamer binding and a molecular subtype or disease stage; it was demonstrated that the aptamer could detect cancer cells in minced tissues of tumors with different molecular subtypes and stages. The low number of patients in the group with the triple-negative subtype (7.6% from all subtypes) made it impossible to assess the specificity of the subtype. Nevertheless, the MDA231 aptamer detected tumor cells in the margins and distant tissues of all molecular subtypes (Supplementary Table S1). For some patients, aptamer binding was observed in tissues from lymph node metastases and distant tissues (7%–54% binding in metastasis and 4%–33% binding in distant tissues). The lymph node metastasis of patients 1, 4, and 7 contained more cancerous cells than the tumor itself. Unfortunately, disease dynamics for these patients are not available.

### 3.3 Aptamer-based CTCs isolation from the blood samples of patients with breast cancer

Dissemination of tumor cells from the primary tumor into the bloodstream is a critical step in breast cancer and tumorigenesis and is considered a precursor of distant metastases (Janni et al., 2016). The MDA231 aptamer selected for highly malignant tumor cell lines might detect CTCs with metastatic potential.

**FIGURE 4:**
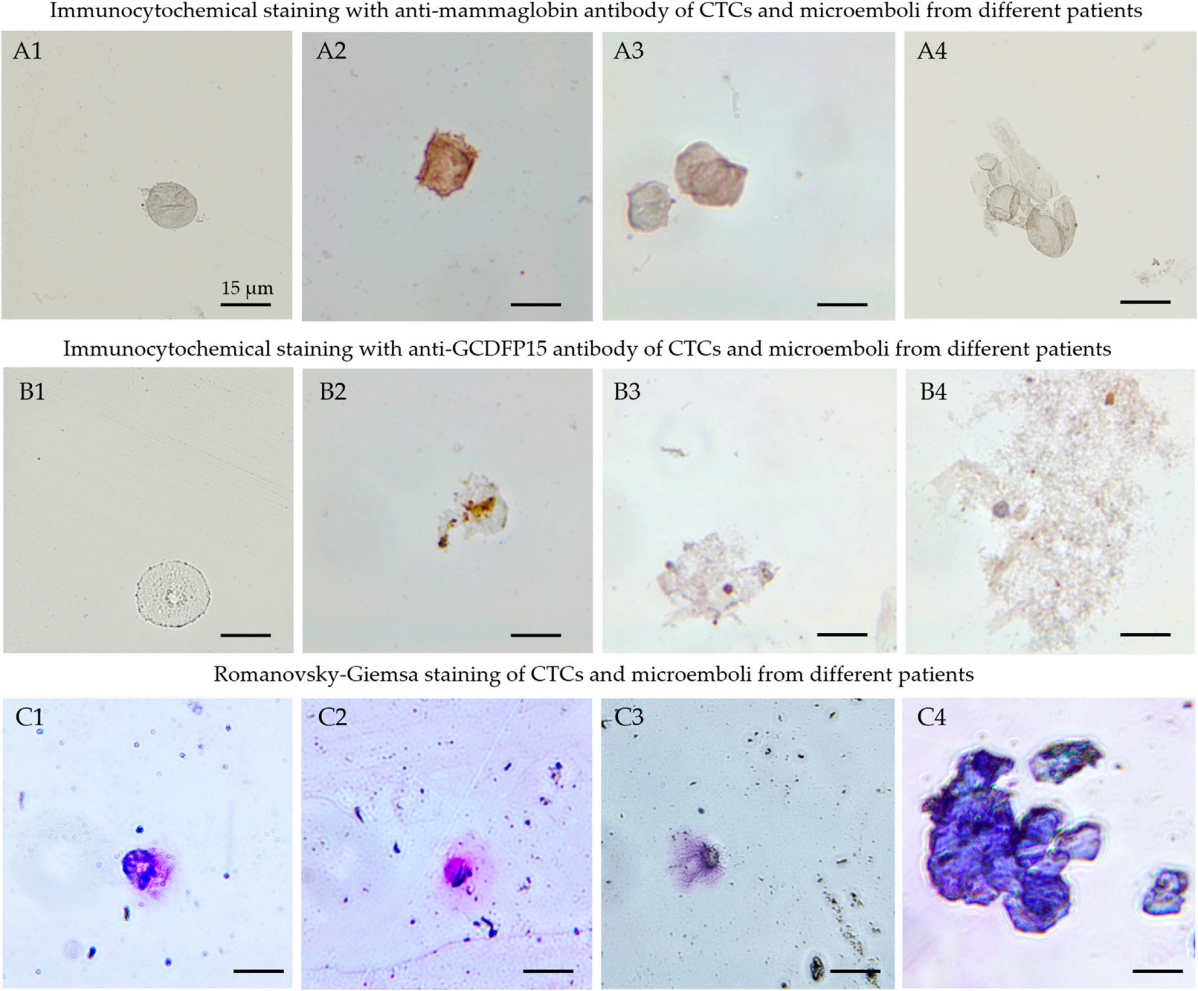
Cytological analyses of the isolated CTCs. Immunocytochemical staining with anti-mammaglobin **(A)**, anti-GCDFP15 antibodies **(B)** of CTCs (A1, A2, B1, B2) and microemboli (A3, A4, B3, B4) from different patients. Morphology of the isolated cells (C1-C3) and microemboli (C4) was confirmed by Romanovsky-Giemsa staining **(C)**.

To test the hypothesis, CTCs and circulating tumor micro-emboli (CTMs) were isolated from the patient’s blood using the protocol described previously (Zamay et al., 2019). CTCs and CTMs were captured with the biotinylated aptamer MDA231 attached to streptavidin-coated magnetic beads and stained with the same Cy3-labeled aptamer. To prove that affinity isolated cells have BC origin, we performed their immune cytochemical analyses with anti-mammaglobin (Figure 4A) and anti-gross cystic disease fluid protein (GCDFP15) antibodies (Figure 4B). Antibodies to these proteins can be used to classify BC and metastatic tumors of breast origin. However, we must note that mammaglobin and GCDFP15 expression is reduced in high-grade and metastatic tumors (Bhargava and Dabbs, 2011; Chivukula and Dabbs, 2011; Willys and Nazaretian, 2013; Darb-Esfahani et al., 2014). CTCs were magnetically captured from the blood of 6 patients with different molecular subtypes of breast cancer (Supplementary Table S2). We have found CTCs in the blood of 5 patients, all of them had GCDFP15-positive cells. Immunocytochemical staining revealed mammaglobin-positive cells in the blood of all five patients with CTCs. At the same time, CTCs only 4 of the expressed mammaglobin mRNA at the detectable range, probably because of a low number of cells or reduced expression of this protein. CTC morphology was confirmed by staining with Romanovsky-Giemsa dye (Figure 4C). Most CTCs had irregular shapes and looked like epithelial cells with nuclei of different sizes and shapes, indicating the aggressive nature of primary cancer (Gascoyne and Shim, 2014).

**FIGURE 5:**
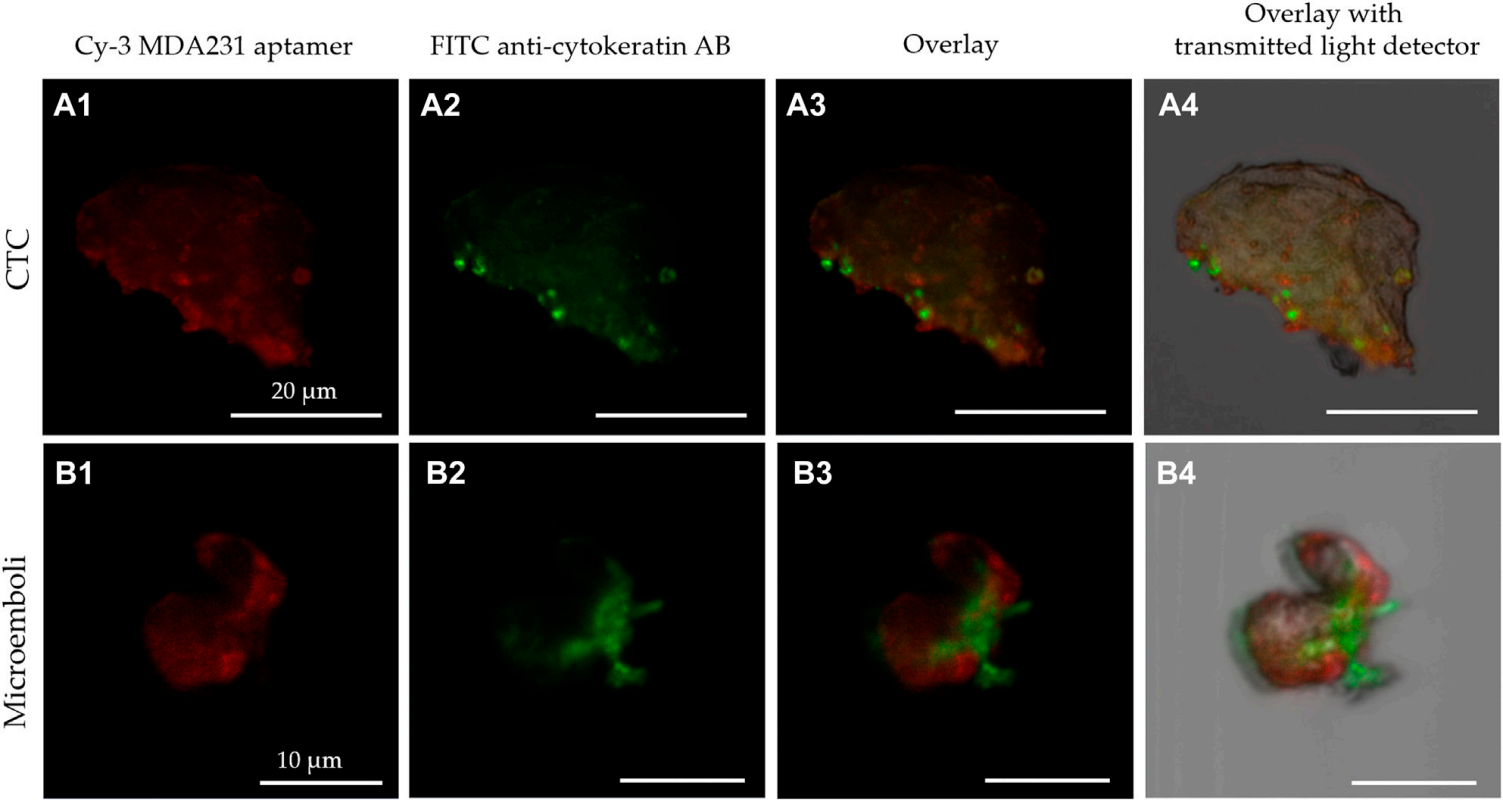
Laser scanning microscopy of CTC **(A)** and microemboli from 2 cells **(B)** isolated from the blood of breast cancer patients stained with Cy-3 MDA231 aptamer (A1, B1), FITC-anti-cytokeratin antibody (A2, B2). Cy-3 MDA231 aptamer and FITC-anti-cytokeratin antibody overlay is represented at the panels A3, B3; overlay with transmitted light detector at – A4, B4.

The results have been validated by the expression of mammaglobin mRNA in CTCs of the same patients. This sensitive method originally has been developed for the analyses of circulating mammaglobin mRNA. Here we applied it to find it in CTCs. CTCs from 4 patients expressed mammaglobin mRNA differently and correlated with the immune cytochemical results (Supplementary Table S2). Thus, this magnetic affinity CTCs capture with MDA 231 aptamer can be used for specific breast cancer CTCs isolation. Blood samples from 27 patients were examined using this approach (Supplementary Table S3; Figure 5). After isolation, CTCs were stained with Cy3-labeled MDA231 (Figures 5A1, B1) and anti-cytokeratin antibody; afterward, smears on glass slides were prepared and analyzed using fluorescence microscopy. CTCs epithelial origin was defined by staining with an anti-cytokeratin antibody (Figures 5A2, B2). We must note that some MDA231 positive cells were cytokeratin negative, which might be evidence of epithelial-mesenchymal transition. Figure 5 presents CTC and CTM isolated by biotinylated MDA231 and stained with the same Cy3-labeled aptamer imaged by laser scanning microscopy. CTC morphology was confirmed by staining with Romanovsky-Giemsa dye.

Seven patients were available for the analyses of blood samples 32 months after the surgery and therapy. It is essential that five patients in remission did not have any CTCs in their blood. However, three patients with metastasis in the liver, lung, and lymph nodes had 2, 6, and 9 cells isolated using MDA231 aptamer, respectively. To assess the prognostic potential of CTCs identification in blood, we correlated CTCs numbers with the following clinical and molecular parameters: expression indicators of estrogen and progesterone receptors, human epidermal growth factor type 2 receptor, proliferative activity of a tumor cell, the degree of tumor differentiation and the histological structure of the tumor process. We did not find any significant correlations.

Five patients were examined 48 months after the surgeries. Two had clinical stabilization of the disease and did not have CTCs in the blood samples. In one patient with continued growth in the postoperative scar, 3 CTCs isolated by MDA 231 were found. Two patients with 4 and 8 CTCs had no clinical data for disease recurrence at the last scheduled examination by an oncologist.

Next, we attempted to identify a potential correlation between CTC numbers and disease stage or molecular subtype. Molecular markers ER, PR, Ki67, and Her-2 were assessed. The distribution of patients according to the stages of the disease was carried out according to the pathological and anatomical conclusions based on the TNM system (7th edition, 2010): stage I—5 (22.7%) patients, stage II—12 (54.6%) patients and stage III—in 5 (22.7%) patients.

The histological grade (G) was determined according to the Nottingham system. According to this classification, 3 features were evaluated: tubule formation, nuclear polymorphism, and mitoses. I degree (low)—3-5 points; II degree (moderate)—6-7 points; III degree (high)—8-9 points. The distribution of patients according to the degree of malignancy: I—18.2%, II—72.7%, III—9.1%.

The expression of estrogen and progesterone receptors was assessed semi-quantitatively using the Allred scoring system. Determination of HER2/neu expression was performed using immunohistochemistry. Result 0.1+—Negative expression, 2+—Questionable, 3+—Positive.

The distribution of patients by molecular biological subtypes of breast cancer was carried out based on the following criteria:

1. Luminal A: estrogen receptor (ER) positive (Allred) and progesterone receptor (RP) (≥6), HER2/neu expression (0–1+), Ki67 < 20%.
2. Luminal B (HER2 negative): estrogen receptor (ER) and/or progesterone receptor (RP) positive (Allred ≤ 5), Ki67 ≥ 20%. Luminal B (HER2 positive): estrogen receptor (ER) and/or progesterone (RP) positive status, HER2 overexpression (3+), Ki67—Any.
3. HER2—Overexpressing breast cancer: overexpression of HER2/neu (3+), lack of expression of steroid hormone receptors, Ki67—Any.
4. Triple-negative breast cancer: no expression of steroid hormone receptors (RE, RP), no expression of HER2/neu (<2+), Ki67—any.

**FIGURE 6:**
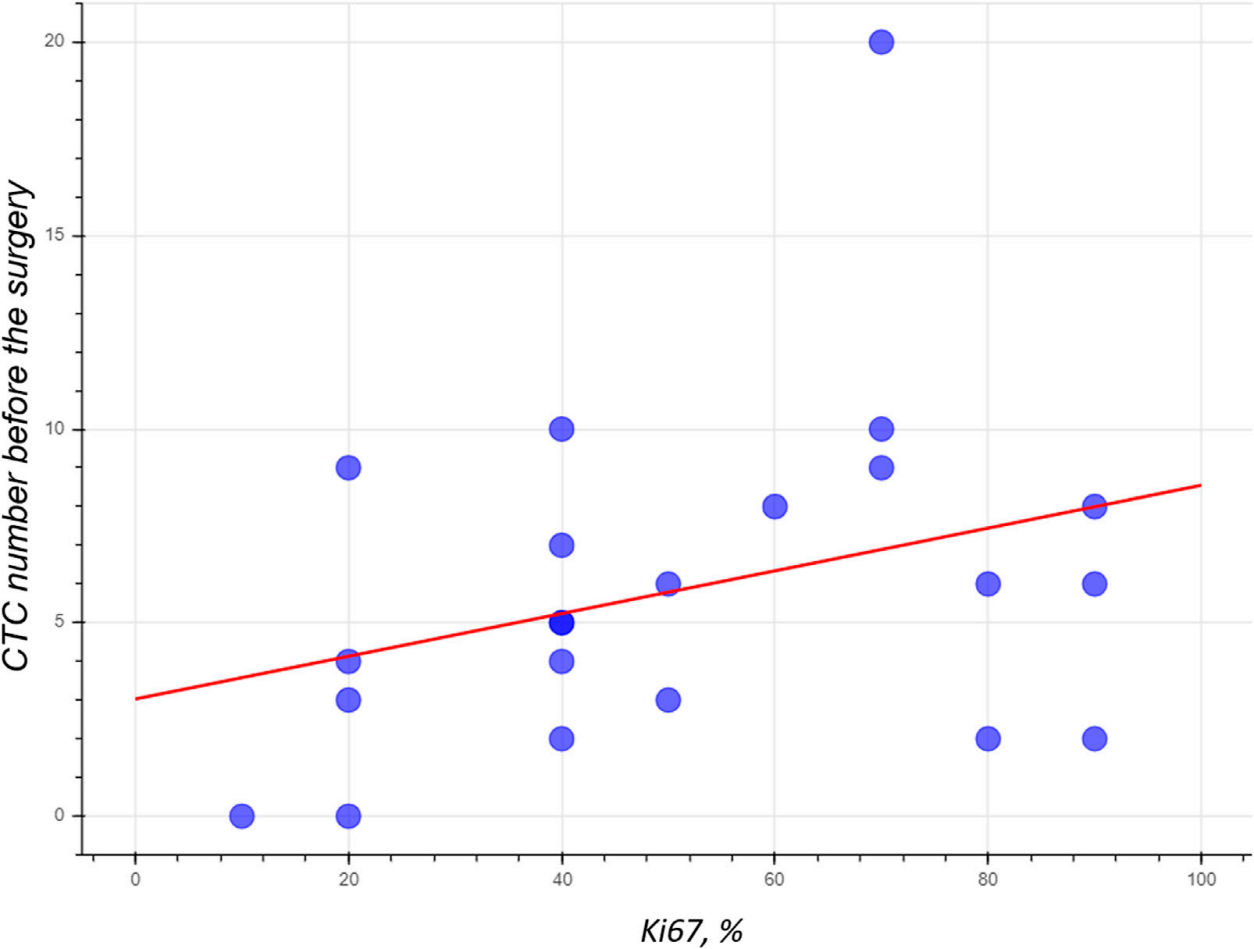
The numbers of CTCs isolated from the blood of BC patient using the MDA231 aptamer have the tendency to depend on Ki67 expression in tumor tissues.

9.1% of patients participating in the study have luminal A subtype 2 (3 patients), 59.1% of patients have luminal B subtype (HER2-negative) (13 patients), 18.2% of patients have luminal B subtype (HER2-positive) (4 patients), 1 patient (4.5%) has HER2 overexpressing, 2 patients (9.1%) are triple-negative. Unfortunately, the small number of patients was insufficient to find reliable patterns. There was no correlation between grade, hormone receptor status, tumor stage, degree of histological differentiation, metastasis, disease progression, and the number of CTCs. We only noticed the tendency towards direct dependence between CTCs number and the percentage of Ki67 expression in tumor tissues R2 = 0.10 (Figure 6). The MDA-MB-231 cell line to which the aptamer was selected originated from malignant cancer with a high proliferative index, and therefore the aptamer could bind CTCs capable of forming the metastasis.

### 3.4 Aptamer targets identification

We showed that aptamer MDA231 binds to cancer cells from BC patients. Thus, it proves that the target of MDA231 is a protein, that is, not downregulated during the transformation of the culture and is not specific to the exact molecular subtype. This, in turn, suggests that the target of MDA231 is one of the essential proteins needed for cell metabolism. We have identified aptamer-associated proteins using the modified AptaBID technique. Protein pull-down experiments were repeated 4 times for each tissue sample taken from 4 patients. All mass spectrometry analyses were made in duplicates or triplicates, and 44 replicates were made. The most prevalent proteins identified in the samples after enrichment with aptamers are vimentin and lumican (Table 1).

**TABLE 1:** The occurrence of confidently identified protein targets candidates in the total amount of samples. Pt 1—Pt 4—Patient numbers. The amounts of analytic replicates are indicated in brackets.

N	ID	Gene	Name	Pt 1 (12)	Pt 2 (14)	Pt 3 (10)	Pt 4 (8)	Total (44)
1	P08670	VIM	Vimentin	3	11	1	1	16
2	P51884	LUM	Lumican	3	11	0	0	14
3	P17661	DES	Desmin	2	9	0	0	11
4	P07355	ANXA2	Annexin A2	4	3	0	1	8
5	P22105	TNXB	Tenascin-X	2	5	0	1	8

## 4 Discussion

BС diagnosis continues to evolve, and new methods are entering medical practice and standard detection protocols used for decades. At present, fluorescent-guided imaging and liquid biopsy are becoming more widely used. Various physical methods allow for improving equipment for tumor marker detection. In contrast, the development of molecular technologies allows searching and investigating of new specific ligands of BC and other oncogenic diseases. So, along with antibodies, DNA- and RNA-aptamers have the potential for diagnostic applications such as real-time visualization or detection of oncogenic proteins in the blood, plasma, or histological tissue sections.

Currently, epithelial cell marker is used in CellSearch^®^, the only Food and Drug Administration-approved method for CTCs detection in breast, prostate, and colorectal cancer patients. However, these criteria do not cover existing needs because it does not allow to detection of cell mesenchymal phenotype (Lozar et al., 2020).

Here we used the aptamer MDA231 selected against the malignant breast cancer cell line MDA-MB-231. Dassie (2012) for the specific breast cancer cell detection in clinical samples. Here we suggested secondary and tertiary structures of the aptamer and proved that it binds with the cells isolated from real clinical samples of BC patients.

It was observed that aptamer MDA231 stains cells from tumor tissue and tumor margin but less efficiently and detects cancer cells in metastatic lymph nodes. Confocal laser microscopy of histological tissue sections proved flow cytometry analysis and showed that the aptamer has specificity for oncogenic cells. Moreover, MDA231 linked with streptavidin magnetic beads isolated CTCs and CTMs with breast cancer origin from the patients’ blood.

Quantitative determination of circulating tumor cells allows us to assess the risk of recurrence or progression of the disease and monitor the treatment’s dynamics and course. The main problem in CTCs detection is transformation during disease development. CTCs lose or get new features and proteins, and so “escape” from a researcher. Aptamer MDA231 stained CTCs in patients’ blood before surgery and 32 and 48 months after it. In this study, we did not aim to find a correlation between BC stage and type and the number of CTCs; however, it was important to know the ability of MDA231 to isolate CTCs during the disease progression. The fact that the MDA231 isolated CTCs within months of BC detection may indicate its specificity for key markers, which do not disappear during cell transformation. Dr. Kuhar’s research group also tried to find correlations between CTCs and clinical parameters for breast cancer; more comprehensive studies must be performed to find any regularities (Lozar et al., 2020).

Two of the five probable target proteins of MDA231 are vimentin and lumican, which participate in BC metastasis. Vimentin is a type III intermediate filament protein; it regulates the EMT of tumor cells, modulates the migration, invasion, and adhesion of tumor cells, exerts regulatory effects on angiogenesis, and may be a potential target for BC therapy (Chen et al., 2021). Moreover, cell surface vimentin is a target for CTCs isolation in various cancer types (Gao et al., 2021).

Lumican is a class II small leucine-rich proteoglycan that regulates cancer proliferation within the tumor microenvironment (Appunni et al., 2021). It regulates estrogen receptors, the expression of matrix macromolecules, and epithelial-to-mesenchymal transition (Karamanou et al., 2020). Reduced expression of lumican is associated with poor outcomes in node-negative BC (Troup et al., 2003).

## 5 Conclusion

The aptamer MDA231 selected against MDA-MB-231 cell culture originated from triple-negative breast cancer and showed specificity for oncogenic proteins in real clinical samples of BC patients with different molecular subtypes. MDA231 has the potential for the detection of BC markers in tissue, margin, and lymph nodes and the isolation of CTCs from blood. Currently, we did not find any significant correlations between tumor characteristics and response to the therapy with the affinity of the aptamer MDA231 to the main tumor node, metastasis, distant tissues, or CTCs/microemboli number in blood samples. However, isolated CTCs can be used for further molecular or immunocytochemical analyses.

## Data Availability

The original contributions presented in the study are included in the article/Supplementary material; statistical analyses are available at the following link: https://drive.google.com/file/d/1VuFlfVztpFpVN949bez6K6chLwUWCBLa/view?usp=sharing; further inquiries can be directed to the corresponding author.
