# Supplementary material for: Monitoring of breast cancer progression via aptamer-based detection of circulating tumor cells in clinical blood samples
Source: Front Mol Biosci. 2023 Jun 8;10:1184285. doi: 10.3389/fmolb.2023.1184285 (PMC10285395; doi:10.3389/fmolb.2023.1184285)
Supplement: Supplementary file 1 [file DataSheet1.DOCX]

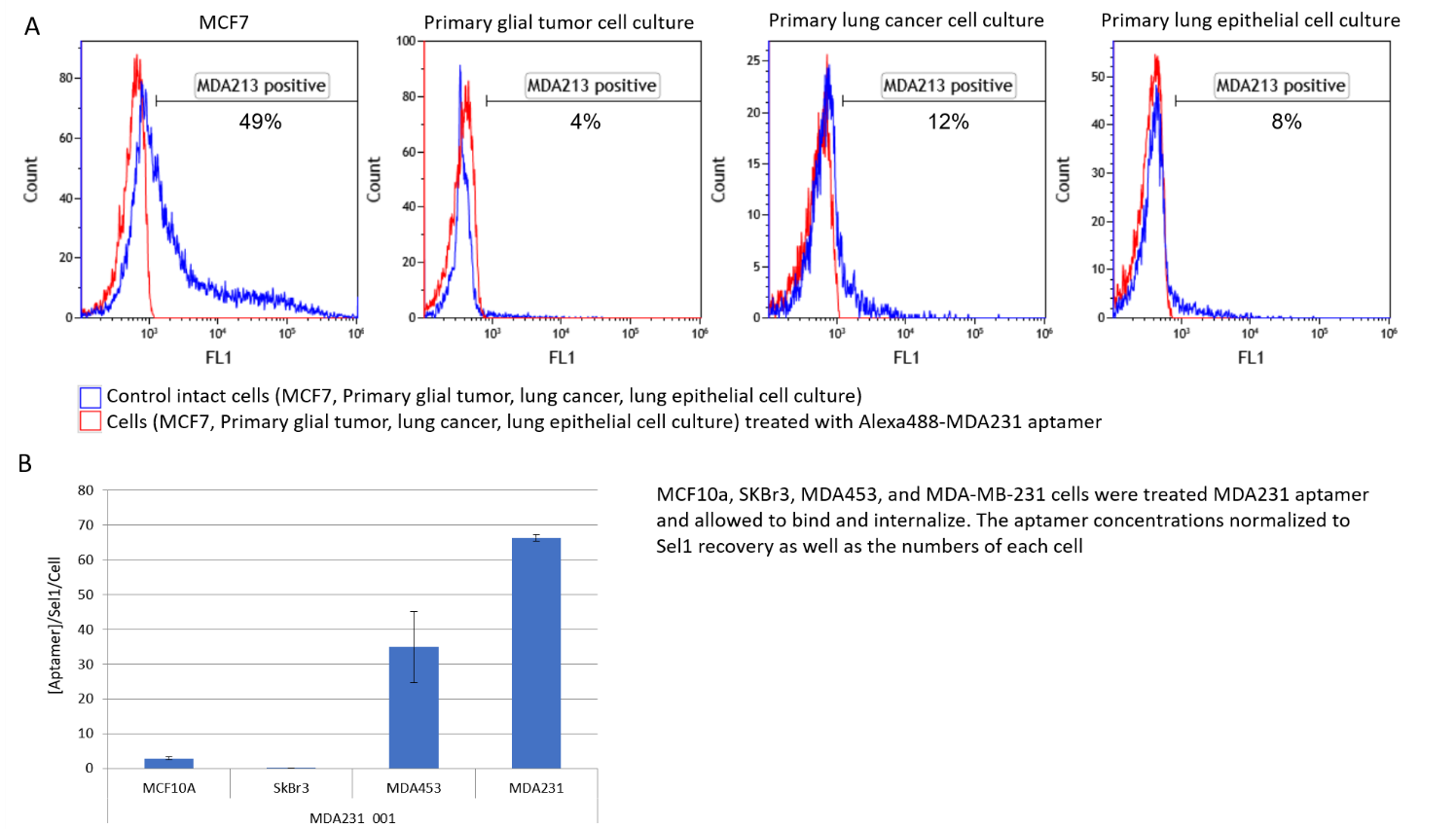


Supplementary Figure S1. MDA231-aptamer specificity analyses. Flow cytometry

Supplementary Table S1. List of breast cancer patients included in the analysis, their age, the type and stage of the disease, and levels of molecular markers HER2, PR, ER, KI67 matched with the percentage of aptamer binding.

| PI* | Histology | Stage / Degree of histological differentiation (G) | Molecular biological subtype | ER, PR, HER2, KI67, % | Cells bound with aptamer, % | | | | Disease dynamics – remission, progression (metastatic sites) |
| --- | --- | --- | --- | --- | --- | --- | --- | --- | --- |
|  |  |  |  |  | T* | M* | DT* | LN* |  |
| 1 | Invasion ductal carcinoma with cellular elements anaplasia | T2N3M0 / 3 | Luminal B (HER2 negative) | ER-5,  PR-0, HER2-0,  KI67-40% | 20 | 13 | 7 | 54 | Progression (Mts. in lymph nodes and liver) |
| 2 | Multicentric growth, infiltrating solid carcinoma. | T2N0M0 / 2 | Luminal B (HER2 negative) | ER-7, PR-7 HER2-0, Ki67-40% | 65 | 9 | 5 | 7 | Remission |
| 3 | Infiltrating solid carcinoma, tubular scirrhous | T1N1M0 / 2 | Luminal A | ER-8,  PR-8, HER2-0, Ki67-20% | 46 | 39 | 33 | 7 | Remission |
| 4 | Ductal carcinoma | T2N1M0 / 2 | Luminal A | ER-4,  PR-5, HER2-0, Ki67-10% | 7 | 4 | 4 | 16 | Remission |
| 5 | Invasive carcinoma | T2N1M0  Relapse  / 2 | Luminal A | ER-8,  PR-8, HER2-1+,  Ki67-10% | 49 | 40 | 10 | ND* | Remission |
| 6 | Infiltrative malignant carcinoma with necrosis,  suppuration, multicentric growth. | T2N3M0 / NA | Triple-negative | ER-0, PR-0,  HER2-0,  Ki67-40% | 18 | 11 | 7 | 37 | ND |
| 7 | Invasive lobular carcinoma withs cirrhous form of growth | T1N0M0 / 3 | Luminal A | ER- 3,  PR - 5,  HER2-0,  Ki-67- 15 % | 27 | 11 | ND | 17 | ND |
| 8 | Invasive carcinoma, solid-type | T2N0M0 / 2 | Luminal B (HER2 positive) | ER-4,  PR-5, Her2-3,  KI67%-70, | 36 | 27 | ND | ND | Remission |
| 9 | Invasive ductal carcinoma | T2N2M0 / 3 | Luminal B (HER2 negative) | ER-8,  PR-7, Her2-1,  KI67%-60, | 26 | 8 | ND | ND | Remission |
| 10 | Invasive carcinoma, with the solid-tubular type of structure | T2N0M0 / 2 | Luminal B (HER2 negative) | ER-8,  PR-8, Her2-0 KI67%-40, | 43 | 17 | ND | ND | Remission |
| 11 | Invasive lobular carcinoma, grade III of therapeutic pathomorphosis | T2N0M0 / 3 | Luminal B (HER2 positive) | ER-3,  PR-4, Her2-3, KI67%-10, | 37 | 11 | ND | ND | Remission |
| 12 | Invasive lobular carcinoma | T1сN0M0 / 1 | Luminal A | ER-8,  PR-8, Her2-1,  KI67%-20, | 38 | 15 | ND | ND | Remission |
| 13 | Mucous cancer | T1аN0M0 / NA | Luminal A | ER-8,  PR-8, Her2-0, KI67%-10, | 90 | 77 | ND | ND | Remission |
| 14 | Invasive ductal carcinoma | T1сN0M0 / 2 | Luminal B (HER2 negative) | ER-8,  PR-8, Her2-1, KI67%-70, | 45 | 14 | ND | ND | Remission |
| 15 | Invasive ductal carcinoma | T2N0M0 / 2 | Luminal B (HER2 negative) | ER-8,  PR-7, Her2-0, KI67%-50, | 32 | 11 | ND | ND | Progression (Mts. in liver and bones) |
| 16 | Invasive ductal carcinoma | T2N0M0 / 2 | Luminal B (HER2 negative) | ER-8,  PR-8, Her2-0, KI67%-30, | 68 | 19 | ND | ND | Remission |
| 17 | Invasive ductal carcinoma | T1bN0M0 / 1 | Luminal A | ER-8,  PR-8, Her2-0, KI67%-10, | 30 | 7 | ND | ND | Remission |
| 18 | Invasive ductal carcinoma | T2N0M0 / 3 | Luminal A | ER-8,  PR-8, Her2-0, KI67%-10, | 47 | 12 | ND | ND | Remission |
| 19 | Invasive ductal carcinoma | T1сN0M0 / 2 | Luminal B (HER2 negative) | ER-8,  PR-4, Her2-0, KI67%-20, | 44 | 10 | ND | ND | Remission |
| 20 | Invasive ductal carcinoma, solid-skirrhotic structure type | T1сN0M0 / 2 | Luminal A | ER-8,  PR-8, Her2-0, KI67%-20, | 87 | 6 | ND | ND | Remission |
| 21 | Invasive ductal carcinoma, solid-skirrhotic structure type | T1сN0M0 / 2 | Luminal B (HER2 negative) | ER-8,  PR-4, Her2-2,  KI67%-30,  FISH – negative | 33 | 31 | ND | ND | Remission |
| 22 | Invasive ductal carcinoma. | T1сN0M0 / 2 | Triple-negative | ER-0,  PR-0, Her2-0,  KI67%-80, | 57 | 6 | ND | ND | Remission |
| 23 | Invasive ductal carcinoma, skirrhotic structure type | T2N0M0 / 2 | Luminal B (HER2 negative) | ER-7,  PR-7, Her2-0, KI67%-30, | 62 | 5 | ND | ND | Remission |
| 24 | Invasive ductal carcinoma, skirrhotic structure type | T2N0M0 / 2 | Luminal A | ER-8,  PR-8, Her2-0, KI67%-10, | 28 | 17 | ND | ND | Remission |
| 25 | Invasive ductal carcinoma, skirrhotic structure type | T1сN0M0 / 3 | Luminal B (HER2 negative) | ER-8,  PR-8, Her2-0, KI67%-30, | 70 | 27 | ND | ND | Remission |
| 26 | Invasive ductal carcinoma, skirrhotic structure type | T1bN0M0 / 2 | Luminal A | ER-8,  PR-8, Her2-0, KI67%-10, | 78 | 15 | ND | ND | Remission |
| 27 | Invasive ductal carcinoma, skirrhotic structure type | T1сN1M0 / 2 | Luminal A | ER-8, PR-8,  Her2-0, KI67%-10, | 8 | 6 | ND | ND | Remission |

*PI - Patient identification number, *T – Tumor, *M – Margin, *DT - Distant tissue, *LN - Lymph node, *ND - No data.

Supplementary Table S2. Verification of breast cancer origin of CTCs isolated from patient’s blood.

| PI* | Diagnosis / Stage / Molecular biological subtype | Hormone, receptor status | Total CTCs amount | Immunocytochemistry  mammaglobin / / GCDFP15 | mRNA mammaglobin expression |
| --- | --- | --- | --- | --- | --- |
| 34 | Cancer mammae sinistrae  IIB (T2N1M0)  Luminal B | ER 8  PR 8  HER2 0  Ki67% 30 | 0 | 0 | - |
| 29 | Cancer mammae dextrae  0 (cTisN0M0) Luminal A | ER 8  PR 7  HER2 0  Ki67% 5 | 26 | 7/6 | 56 |
| 30 | Cancer mammae dextrae  IV (сT4N0M1)  Mts lungs, bones  Triple negative | ER 0  PR 0  HER2 0  Ki67% 60 | 54 | 26/13 | 630 |
| 31 | Cancer mammae sinistrae  IIA (сT2N0M0)  Triple negative | ER 0  PR 0  HER2 0  Ki67% 60 | 17 | 2/3 | - |
| 32 | Cancer mammae sinistrae  IIA (сT2N0M0)  HER2 hyperexpression | ER 0  PR 0  HER2 3+++  Ki67% 60 | 41 | 13/9 | 265 |
| 33 | Cancer mammae dextrae  IIB (сT2N1M0) | ER 8  PR 6  HER2 0  Ki67% 60 | 24 | 6/0 | 1 |
|  |  |  |  |  |  |
|  |  |  |  |  |  |

Supplementary Table S3. The list of BC patients and the number of CTCs and CTMs isolated from their blood using the MDA231 aptamer.

| PI | Degree of histological differentiation (G) | | Age | Stage | ER, PR, KI67%, Her-2 | CTC/ CTM amount  Before the surgery | CTC/ CTM amount  32 months after the surgery | Disease progression at 32 months | CTC/ CTM amount  48 months after the surgery | Disease progression at 48 months |
| --- | --- | --- | --- | --- | --- | --- | --- | --- | --- | --- |
| 34 | 2  (therapeutic pathomorphosis grade 3) | 50 | | ypT3N0M0 | ER-2, PR-0, KI67%-70, Her2-3 | 20/1 | ND* | ND | ND | ND |
| 35 | 3 (sarcoma) | 54 | | pT3N1M0 | ER-0, PR-0, KI67%-90, Her2-1 | 6/0 | ND | Mts. to the right lung and | ND | Continued mts. growth to the right lung and brain |
| 36 | 1 | 43 | | pT1cN1M0 | ER-7, PR-6, KI67%-40, Her2-1 | 5/0 | ND | ND | 8/0 | ND |
| 37 | 2 | 44 | | pT2N1M0 | ER-8, PR-8, KI67%-40,Her2-0 | 10/3 | ND | ND | ND | ND |
| 38 | 2 | 38 | | pT1сN0M0 | ER-8, PR-4, KI67%-20, Her2-2 | 9/0 | 0/0 | Remission | 0/0 | Remission |
| 39 | 2 | 35 | | pT1N1M0 | ER-8, PR-3, KI67%-70, Her2-2 | 10/3 | ND | ND | ND | Mts. in liver |
| 40 | 2 | 60 | | pT1сN3M0 | ER-6, PR-5, KI67%-70, Her2-1 | 9/0 | 0/0 | Remission | ND | ND |
| 41 | 2 | 63 | | pT2N0M0 | ER-8, PR-8, KI67%-50, Her2-3 | 6/1 | 6/0 | Mts. in liver | ND | Stabilization |
| 42 | 2 | 51 | | pT1сN0M0 | ER-8, PR-4, KI67%-40,Her2-1 | 2/0 | ND | ND | ND | ND |
| 43 | 3 | 64 | | pT4N2M0 | ER-8, PR-8, KI67%-60, Her2-0 | 8/1 | ND | Mts. in lung, death 16 months after the surgery | - | - |
| 44 | 2 | 68 | | pT1сN1M0 | ER-8, PR-7, KI67%-20, Her2-1 | 3/0 | 0/0 | Remission | 4/0 | ND |
| 45 | 2 | 66 | | pT2N1M0 | ER-7, PR-5, KI67%-40, Her2-3 | 4/0 | 9/0 | Mts. in lung, bones | - | Death 16 months after the surgery |
| 46 | 2 | 67 | | pT1сN0M0 | ER-8, PR-5, KI67%-40, Her2-0 | 7/0 | ND | ND | ND | ND |
| 47 | 2 | 60 | | pT2N0M0 | ER-8, PR-8, KI67%-90, Her2-0 | 2/0 | ND | ND | ND | ND |
| 48 | 2 (therapeutic pathomorphosis grade 3) | 44 | | ypT3N1M0 | ER-8, PR-5, KI67%-20, Her2-1 | 4/2 | 2/0 | Mts. in lymph nodes | ND | Remission |
| 49 | 2 (therapeutic pathomorphosis grade 3) | 47 | | ypT4N0M0 | ER-8, PR-7, KI67%-50, Her2-3 | 3/0 | ND | Mts. in postoperative scarp | 3/0 | Continued mts. growth in postoperative scarp |
| 50 | 1 | 68 | | pT2N0M0 | ER-8, PR-7, KI67%-20, Her2-0 | 0 | 0/0 | Remission | ND | ND |
| 51 | 1 | 45 | | pT1сN0M0 | ER-8, PR-8, KI67%-40, Her2-1 | 2/0 | 0/0 | Remission | ND | ND |
| 52 | 2 | 45 | | pT1сN0M0 | ER-8, PR-8, KI67%-40, Her2-0 | 5/1 | ND | ND | 0/0 | Remission |
| 53 | 1 | 46 | | pT3N0M0 | ER-8, PR-6, KI67%-10, Her2-0 | 0/0 | ND | ND | ND | ND |
| 54 | 2  (complete therapeutic pathomorphosis) | 45 | | ypT3N0M0 | ER-0, PR-0, KI67%-90, Her2-0 | 8/1 | ND | ND | ND | ND |
| 55 | 2 | 79 | | pT1сN1M0 | ER-8, PR-8, KI67%-80, Her2-2 | 6/2 | ND | ND | ND | ND |
